# Transthoracic Echocardiography as a Tool for Early Detection of Atrial Fibrillation in Patients Receiving Ibrutinib

**DOI:** 10.3390/life16020324

**Published:** 2026-02-13

**Authors:** Vittoria Gammaldi, Martina Pucci, Francesca La Rocca, Pasquale Megaro, Daniele Paoletta, Mariateresa Pontoriero, Luca Maria Capece, Roberto Luise, Marina Iacono, Roberta Esposito

**Affiliations:** 1 Department of Clinical Medicine and Surgery, Federico II University Hospital, 80131 Naples, Italy; 2 Department of Clinical and Experimental Medicine, University of Catania, 95122 Catania, Italy

**Keywords:** chronic lymphocytic leukemia, ibrutinib, Bruton’s tyrosine kinase inhibitors, atrial fibrillation, cardio-oncology, left atrial strain, speckle-tracking echocardiography, atrial remodeling, cardiovascular toxicity

## Abstract

**Background**: Bruton’s tyrosine kinase inhibitors, particularly ibrutinib, have improved outcomes in patients with chronic lymphocytic leukemia but are associated with an increased risk of atrial fibrillation. The early identification of patients with increased susceptibility to atrial fibrillation remains a major challenge in cardio-oncology. **Methods**: This prospective pilot study included 45 patients with chronic lymphocytic leukemia treated with ibrutinib. All patients underwent comprehensive transthoracic echocardiography at baseline and after 6 months. Left atrial structure and function were assessed, with particular emphasis on speckle-tracking-derived left atrial strain parameters, including peak atrial longitudinal strain and peak atrial contraction strain. **Results**: At follow-up, a modest but significant increase in indexed left atrial volume was observed, while left atrial functional parameters remained stable. Patients who developed atrial fibrillation showed significantly lower baseline Peak Atrial Contraction Strain values compared with those who remained in sinus rhythm, whereas no significant differences in Peak Atrial Longitudinal Strain were detected. **Conclusions**: Ibrutinib-related atrial fibrillation appears to be driven primarily by pre-existing atrial vulnerability rather than early drug-induced atrial dysfunction. The baseline impairment of left atrial contractile function may represent a candidate echocardiographic marker of atrial functional vulnerability and may inform cardiovascular surveillance and monitoring strategies in patients treated with ibrutinib.

## 1. Introduction

The expanding use of targeted therapies in hematologic malignancies has reshaped survival expectations while increasingly positioning cardiovascular toxicity as a key determinant of treatment tolerability and long-term outcomes. In chronic lymphocytic leukemia (CLL), Bruton’s tyrosine kinase (BTK) inhibition represents a cornerstone of modern therapy, yet it is accompanied by a clinically meaningful burden of cardiovascular adverse events. CLL is the most common adult leukemia in Western countries and typically affects older individuals, a population in whom baseline cardiovascular comorbidity is prevalent and may amplify the clinical impact of therapy-related cardiac complications [1,2].

Ibrutinib, a first-generation irreversible BTK inhibitor, has demonstrated substantial efficacy across treatment lines and is widely used in CLL [3,4,5,6]. Nevertheless, its cardiovascular safety profile has emerged as a major challenge in cardio-oncology practice. Atrial fibrillation (AF) is the most frequently reported arrhythmia during ibrutinib exposure and represents a leading cause of treatment modification or discontinuation in real-world cohorts, often exceeding discontinuations due to disease progression [7]. The incidence of AF increases over time, with rates ranging from 3 to 7% early in therapy and rising with longer follow-up [8,9]. In addition to toarrhythmia, ibrutinib has been associated with incident or worsening hypertension, which may contribute to an adverse cardiovascular trajectory during prolonged therapy [10,11]. Although less common, serious ventricular arrhythmias and sudden death have also been described, underscoring the need for structured cardiovascular surveillance in susceptible patients [12].

The management of ibrutinib-related atrial fibrillation (IRAF) is uniquely complex because arrhythmia-related thromboembolic risk must be balanced against an increased bleeding propensity. Ibrutinib exerts antiplatelet effects through the inhibition of BTK-dependent and related platelet signaling pathways, including TEC, thereby increasing bleeding risk and complicating the use of anticoagulants and antiplatelet agents frequently required in this older, comorbidity-rich population [13]. As a result, IRAF not only affects cardiovascular outcomes but may also compromise oncologic continuity of care and therapeutic efficacy through dose reduction or discontinuation, making it a prototypical cardio-oncology toxicity in which prevention and risk stratification have direct oncologic consequences [7,14].

Mechanistically, IRAF appears to reflect both atrial substrate vulnerability and drug-related triggers. While ibrutinib achieves its therapeutic benefit by disrupting B-cell receptor signaling, its kinase inhibition is not fully selective, and off-target effects have been implicated in atrial electrophysiological instability and structural remodeling [15,16]. Experimental data support the involvement of signaling pathways linked to myocardial stress responses and fibrosis, including PI3K–Akt axis perturbation and the inhibition of C-terminal Src kinase (CSK), which has been associated with atrial enlargement, fibrosis, and inflammation in preclinical models, paralleling features observed with ibrutinib exposure [17]. Clinically, IRAF often occurs in patients with conventional AF risk factors—older age, hypertension, structural heart disease, and other comorbidities—suggesting that ibrutinib may act as a precipitating factor in those with pre-existing atrial vulnerability [16,18]. Together, these observations support a “two-hit” framework in which therapy-related molecular effects interact with baseline atrial remodeling to promote arrhythmogenesis.

From a cardiovascular standpoint, AF is tightly linked to left atrial (LA) remodeling, characterized by chamber dilation, fibrosis, and impaired reservoir and contractile function. Transthoracic echocardiography (TTE) provides a non-invasive method to characterize this substrate and is particularly suited for cardio-oncology pathways aimed at anticipating and preventing treatment-related toxicity, as emphasized by current international cardio-oncology guidelines. LA volume index is an established marker of chronic remodeling, and standardized chamber quantification is recommended by echocardiography guidelines [19].

However, structural changes may be preceded by functional impairment, and LA strain assessed using speckle-tracking echocardiography has emerged as a sensitive measure of atrial myocardial deformation with prognostic value for incident AF across multiple clinical contexts [20,21]. In addition, LA strain may detect subclinical atrial dysfunction before overt enlargement, potentially improving risk discrimination beyond traditional parameters [20,21].

In the setting of ibrutinib therapy, the early characterization of patients with heightened atrial vulnerability could support tailored cardio-oncology strategies, including intensified rhythm surveillance, the optimization of modifiable risk factors, and individualized antithrombotic planning. Preliminary evidence suggests that baseline LA strain parameters—particularly reduced peak atrial longitudinal strain (PALS) and peak atrial contraction strain (PACS)—may be associated with the subsequent development of IRAF, supporting their potential role as exploratory markers of atrial vulnerability [22]. Accordingly, the present study evaluates echocardiographic indices of LA structure and function, with an emphasis on LA strain analysis, to explore echocardiographic features associated with IRAF in patients treated with ibrutinib. By refining baseline risk stratification, this approach aims to inform personalized cardiovascular surveillance and management strategies, ultimately improving cardiac safety while preserving continuity and efficacy of oncologic therapy.

## 2. Materials and Methods

### 2.1. Study Design and Population

This prospective pilot study enrolled consecutive patients with chronic lymphocytic leukemia (CLL) referred to our cardio-oncology clinic between 2022 and 2025 for cardiovascular evaluation prior to initiation of Bruton’s tyrosine kinase (BTK) inhibitor therapy. The primary objective was to identify echocardiographic parameters associated with an increased risk of treatment-related atrial fibrillation (AF).

A total of 45 patients were included (mean age 72.3 ± 9.3 years; 63.3% female). All patients underwent comprehensive transthoracic echocardiography at baseline (before therapy initiation) and at 6-month follow-up.

### 2.2. Cardiovascular Assessment

The CV evaluation included a complete patient history (comorbidities, concomitant medications, CVAEs, family history of cardiovascular disease), physical examination, blood pressure measurement, 12-lead ECG and 2D transthoracic echocardiography, as recommended by current ESC cardio-oncology guidelines for baseline cardiovascular assessment in patients undergoing potentially cardiotoxic cancer therapies.

### 2.3. Echocardiographic Acquisition and Analysis

TTE examinations were performed using a Vivid E95 ultrasound system (GE Healthcare, Horten, Norway) equipped with a 2.5 MHz phased-array transducer, in accordance with American Society of Echocardiography (ASE) and European Association of Cardiovascular Imaging (EACVI) recommendations [23].

Standard measurements included left ventricular ejection fraction (LVEF), left ventricular mass indexed to height ^2.7^ (LVMi), global longitudinal strain (GLS), and left ventricular diastolic function assessed by transmitral Doppler flow and tissue Doppler imaging of septal and lateral mitral annular velocities. Particular emphasis was placed on left atrial (LA) size and function. All measurements were obtained from three consecutive cardiac cycles of optimal image quality at frame rates between 40 and 80 frames/s.

LA volume was measured at end-ventricular systole using the modified Simpson’s biplane method from apical four- and two-chamber views. LA volume was indexed to body surface area (LAVi), and values > 34 mL/m^2^ were considered indicative of LA enlargement [24,25,26,27,28].

LA strain analysis was performed using speckle-tracking echocardiography on apical four- and two-chamber views. The LA endocardial border was manually traced, excluding pulmonary vein ostia and the left atrial appendage. The software automatically subdivided the atrial wall into six segments per view, generating segmental and global strain curves [20]. Peak atrial longitudinal strain (PALS) and peak atrial contraction strain (PACS) were derived as measures of LA reservoir and contractile function, respectively.

**Figure 1:**
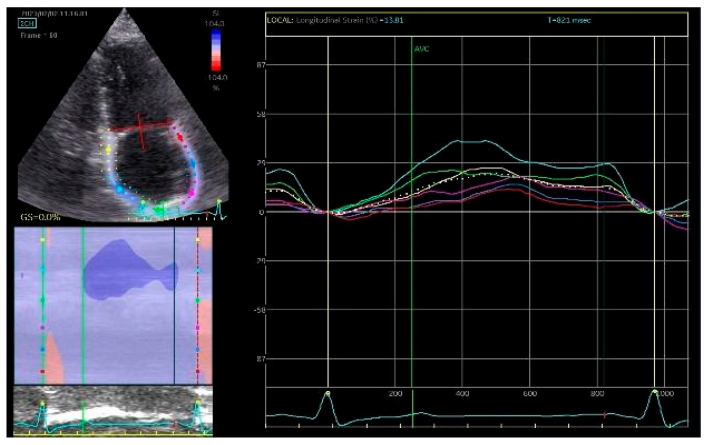
Assessment of LA strain in apical 2-chamber section.

**Figure 2:**
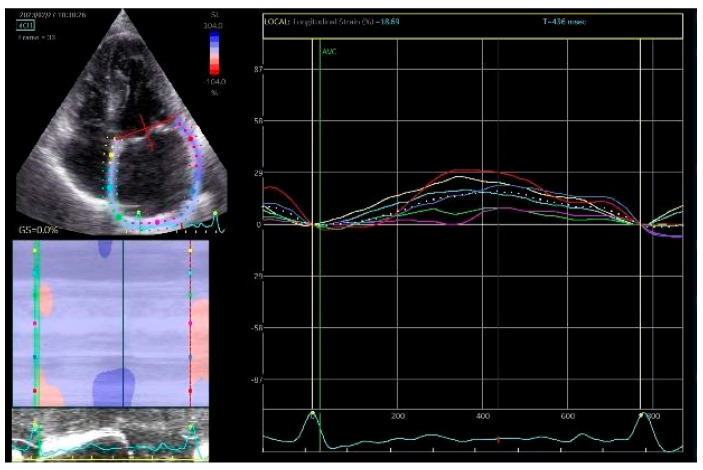
Assessment of LA strain in apical 4-chamber section.

Two principal parameters of LA longitudinal strain are derived. PALS reflects LA reservoir function and is measured at the end of ventricular systole. In healthy individuals, PALS values are typically greater than 40%, whereas reduced PALS has been associated with structural remodeling and fibrosis of the LA myocardium, particularly in patients with atrial fibrillation [29]. PACS is measured immediately before atrial contraction and represents the active contractile contribution of the LA to LV filling (Figure 1 and Figure 2).

### 2.4. Study Endpoints

**Primary endpoints:**

- Occurrence of IRAF during follow-up.

**Secondary endpoints:**

- Changes in echocardiographic parameters between baseline and 6-month follow-up, including:
  ○ LAVi;
  ○ Left atrial strain parameters: PALS, PACS;
  ○ Left ventricular systolic and diastolic function indices.

### 2.5. Statistical Analysis

Statistical analysis was performed using SPSS software (version 12.0; SPSS Inc., Chicago, IL, USA). Continuous variables are expressed as mean ± standard deviation, and categorical variables as numbers (percentage).

Normality of distribution was assessed using Shapiro–Wilk and Kolmogorov–Smirnov tests. As variables did not follow a normal distribution, non-parametric tests were applied. Paired comparisons between baseline and follow-up were performed using the Wilcoxon signed-rank test. Comparisons between patients who developed AF and those who remained in sinus rhythm were conducted using the Mann–Whitney U test. Categorical variables were compared using χ^2^ or Fisher’s exact test, as appropriate. A two-tailed *p*-value < 0.05 was considered statistically significant.

## 3. Results

### 3.1. Clinical Characteristics

No significant differences were observed in baseline clinical characteristics between baseline and 6-month follow-up, including age, body mass index, blood pressure, prevalence of diabetes, or cardiovascular disease. A modest but statistically significant reduction in heart rate was observed at follow-up (69.8 ± 14.2 vs. 66.2 ± 11.3 bpm; *p* = 0.045) (Table 1).

### 3.2. Echocardiographic Findings at Follow-Up

A comparison of echocardiographic parameters between baseline and 6-month follow-up demonstrated the overall stability of left ventricular systolic and diastolic function. LVEF, GLS, pulmonary artery systolic pressure, and E/e′ ratio showed no significant changes over time. A significant increase in left atrial volume index was observed at follow-up (31.5 ± 8.0 vs. 34.3 ± 10.0 mL/m^2^; *p* = 0.015), consistent with early atrial remodeling. In contrast, indices of LA function remained stable, with no significant changes in PALS or PACS values in either apical four- or two-chamber views (Table 2).

### 3.3. Atrial Fibrillation Subgroup Analysis

During the 6-month follow-up, atrial fibrillation occurred in 7 out of 45 patients (15%). All cases were observed in patients treated with ibrutinib. At baseline, all patients were in sinus rhythm.

A comparative subgroup analysis was performed between patients who subsequently developed atrial fibrillation (AF group, *n* = 7) and those who remained in sinus rhythm throughout follow-up (sinus rhythm group, *n* = 38). Baseline clinical characteristics and echocardiographic parameters of left atrial structure and function were evaluated to explore differences associated with the occurrence of atrial fibrillation.

No statistically significant differences were observed between groups with respect to baseline cardiovascular comorbidities, including smoking status, diabetes mellitus, arterial hypertension, dyslipidemia, peripheral vascular disease, or chronic coronary syndrome.

With regard to left atrial structure, baseline left atrial volume index did not differ significantly between groups, although numerically higher values were observed in patients who developed atrial fibrillation (36.95 ± 6.98 vs. 31.56 ± 8.49 mL/mq; *p* = 0.116).

Regarding left atrial function, patients who developed atrial fibrillation exhibited significantly lower baseline peak atrial contraction strain (PACS) values in the apical four-chamber view compared with patients who remained in sinus rhythm (9.75 ± 5.99% vs. 15.72 ± 6.00%; *p* = 0.027). A similar trend toward lower PACS values was observed in the apical two-chamber view, although this difference did not reach statistical significance (10.18 ± 7.37% vs. 16.37 ± 7.77%; *p* = 0.072).

No statistically significant differences were observed between groups for peak atrial longitudinal strain (PALS) in either apical four- or two-chamber views, although numerically lower values were noted in patients who subsequently developed atrial fibrillation. Baseline left atrial volume index did not differ significantly between the two groups.

These findings indicate that patients who developed atrial fibrillation during follow-up were characterized by a baseline impairment of left atrial contractile function, as reflected by reduced PACS values, prior to the initiation of therapy. Given the observational design and limited sample size, these associations should be interpreted as exploratory and hypothesis-generating (Table 3).

## 4. Discussion

The widespread adoption of targeted therapies has fundamentally altered outcomes in hematologic malignancies, while simultaneously shifting attention toward cardiovascular toxicity as a major determinant of treatment tolerability and continuity. Chronic lymphocytic leukemia (CLL), a disease predominantly affecting older individuals with a high burden of cardiovascular comorbidity, exemplifies this evolving cardio-oncology paradigm [30,31]. Among targeted agents, Bruton’s tyrosine kinase (BTK) inhibitors—and particularly ibrutinib—have emerged as highly effective first-line therapies, yet their use is complicated by an increased incidence of atrial fibrillation (AF), a toxicity with direct implications for both cardiovascular and oncologic outcomes [3,5,32,33].

In this prospective pilot study, we sought to identify echocardiographic markers capable of improving cardiovascular risk stratification in patients with CLL undergoing BTK inhibitor therapy. Our principal findings can be summarized as follows: (1) short-term BTK inhibitor therapy was associated with mild structural left atrial remodeling, reflected by a modest increase in indexed left atrial volume; (2) left atrial functional parameters assessed using speckle-tracking echocardiography remained preserved at follow-up; and (3) patients who developed AF exhibited impaired baseline left atrial contractile function, as evidenced by reduced peak atrial contraction strain (PACS), prior to initiation of therapy.

The observation of preserved atrial strain despite mild atrial enlargement is particularly relevant from a cardio-oncology perspective. Atrial remodeling is a dynamic process in which functional impairment may precede overt structural changes, especially in the early phases of atrial disease. Although ibrutinib exposure has consistently been associated with an increased incidence of AF in both clinical trials and real-world cohorts [7,8,16], our findings suggest that the drug does not induce overt short-term atrial myocardial dysfunction. Instead, AF appears to emerge in the context of a pre-existing vulnerable atrial substrate, with therapy acting as a trigger rather than a primary toxic insult. This interpretation is supported by the significantly lower baseline PACS observed in patients who subsequently developed AF, suggesting reduced atrial contractile reserve prior to BTK inhibitor exposure. PACS reflects the active contractile contribution of the left atrium to ventricular filling and may therefore capture subclinical atrial functional impairment even in the absence of significant atrial enlargement.

These results reinforce a “substrate–trigger” model of ibrutinib-related atrial fibrillation (IRAF), in which pre-existing atrial structural and functional abnormalities interact with therapy-related molecular effects to promote arrhythmogenesis. Experimental and translational studies have demonstrated that ibrutinib inhibits multiple kinases beyond BTK, including TEC and C-terminal Src kinase (CSK), with downstream effects on intracellular signaling pathways involved in atrial fibrosis, inflammation, and electrical instability [15,17,34,35]. Notably, the inhibition of CSK has been shown to induce atrial enlargement, fibrosis, and increased AF susceptibility in preclinical models, closely resembling the phenotype observed in patients treated with ibrutinib [17].

Importantly, the absence of deterioration in left atrial strain at follow-up suggests that ibrutinib does not exert a progressive or directly toxic effect on atrial myocardial mechanics over the short term. At first glance, these findings may appear to differ from previous reports describing impaired atrial function in patients treated with BTK inhibitors; however, such differences are largely explained by variations in the study design, timing of echocardiographic assessment, and patient population. Prior studies often evaluated atrial structure and function in patients with established AF or after longer durations of therapy, whereas the present study focused on baseline echocardiographic assessment prior to treatment initiation and short-term follow-up. Accordingly, our findings should be interpreted as complementary rather than contradictory to the existing literature, providing insight into early atrial vulnerability rather than advanced remodeling. This observation aligns with prior echocardiographic studies showing that functional atrial impairment may precede overt structural remodeling and may represent an early marker of AF susceptibility rather than a consequence of arrhythmia or drug toxicity [20,21,36]. From a clinical standpoint, these findings support continued therapy in selected patients, provided that appropriate cardiovascular surveillance is implemented.

Moreover, the availability of fixed-duration treatment strategies incorporating BTK inhibitors—either as monotherapy or in combination regimens—adds an important dimension to cardiovascular risk–benefit assessment. In this context, the preservation of atrial function observed in our cohort may be particularly reassuring, as it suggests that, even in patients at increased arrhythmic risk, exposure to BTK inhibition over a predefined treatment window may be feasible when accompanied by structured cardiovascular monitoring and risk factor optimization. These data may therefore support a broader consideration of fixed-duration BTK inhibitor-based regimens in appropriately selected patients, potentially limiting cumulative cardiovascular exposure while preserving oncologic efficacy.

Notably, all patients in our cohort underwent a systematic optimization of cardiovascular risk factors throughout follow-up. This likely contributed to the preservation of atrial function observed and underscores the importance of multidisciplinary cardio-oncology management. Hypertension, obesity, obstructive sleep apnea, anemia, endocrine disorders, and underlying cardiac or pulmonary disease are well-established contributors to atrial remodeling and AF risk and are highly prevalent in patients with CLL [37,38]. Their correction represents a cornerstone of contemporary AF prevention strategies and may be particularly impactful in patients exposed to BTK inhibitors. Within this framework, echocardiographic assessment of the left atrial structure and function should be viewed as complementary to clinical risk profiling rather than a substitute for it.

From a practical cardio-oncology perspective, our data suggest that the baseline assessment of left atrial contractile function—particularly PACS—may provide incremental information beyond traditional clinical risk factors. Previous studies have demonstrated the predictive role of left atrial strain parameters for incident AF across diverse populations [20,21,36], and emerging evidence supports their utility in identifying patients at increased risk of IRAF [20]. However, given the exploratory nature of the present study, these associations should be interpreted as hypothesis-generating. The incorporation of advanced echocardiographic markers into pre-treatment cardiovascular evaluation may therefore enable personalized monitoring strategies, guide rhythm surveillance, and inform antithrombotic decision-making.

## 5. Limitations

Several limitations of the present study should be acknowledged. First, this was a single-center pilot study with a relatively small sample size, which limits statistical power and precludes extensive multivariable adjustment. Accordingly, the observed associations should be interpreted as hypothesis-generating rather than confirmatory.

Second, given the small number of events, multivariable modeling was not performed in order to avoid model overfitting and unstable estimates. While this approach limits the ability to assess the independent contribution of individual clinical and echocardiographic variables, it was considered methodologically appropriate in the context of an exploratory pilot study.

Third, the duration of follow-up was limited to six months. Although this time frame captures the period of highest incidence of ibrutinib-related atrial fibrillation, it does not allow the assessment of longer-term atrial remodeling cumulative cardiovascular toxicity or late arrhythmic events that may occur with prolonged exposure to BTK inhibitors.

Finally, biomarkers potentially relevant to atrial fibrillation risk, including inflammatory markers such as high-sensitivity C-reactive protein and systematic data on bleeding events, were not uniformly available and could therefore not be incorporated into the analysis. The absence of these variables may have limited a more comprehensive evaluation of clinical and biological contributors to arrhythmic risk.

Future larger, multicenter prospective studies with longer follow-up and integrated clinical, imaging, and biomarker assessment are warranted to validate these findings and to further clarify the role of left atrial strain in the cardiovascular evaluation of patients receiving Bruton’s tyrosine kinase inhibitors.

## 6. Conclusions

In this prospective pilot study, ibrutinib therapy was associated with early structural left atrial remodeling without evidence of short-term deterioration in atrial myocardial function. Importantly, patients who developed atrial fibrillation exhibited impaired baseline left atrial contractile function, as reflected by reduced PACS, prior to treatment initiation.

These findings support the hypothesis that ibrutinib-related atrial fibrillation occurs in the setting of pre-existing atrial vulnerability, with Bruton’s tyrosine kinase inhibitor therapy acting as a precipitating factor rather than a direct atrial myocardial toxin. In this context, the baseline assessment of left atrial strain, particularly PACS, may represent a candidate marker of atrial functional impairment and may help refine cardiovascular evaluation in patients with chronic lymphocytic leukemia considered for ibrutinib therapy.

The integration of advanced echocardiographic markers into pre-treatment cardiovascular evaluation, together with the optimization of modifiable risk factors and close cardio-oncology collaboration, may support more individualized surveillance strategies and arrhythmic risk management, potentially facilitating the continuity of effective oncologic therapy. Larger prospective studies are needed to confirm these observations and to better define the clinical role of left atrial strain-guided approaches in patients receiving BTK inhibitors.

## Figures and Tables

**Table 1:** Comparison between the variables at baseline and after 6 months.

Anthropometric Variables	Baseline	6-Months Follow-Up	*p*-Value
BMI (kg/m^2^)	25.08 ± 3.31	24.94 ± 3.36	0.577
SBP (mmHg)	128.88 ± 14.25	128.33 ± 16.68	0.835
DBS (mmHg)	74.93 ± 11.33	73.00 ± 10.73	0.277
**HR (bpm)**	**69.77 ± 14.21**	**66.15 ± 11.25**	**0.045**

Abbreviations: BMI, Body Mass Index; SBP, Systolic Blood Pressure, Diastolic Blood Pressure, HR: Heart Rate.

**Table 2:** Comparison between the variables at baseline and after 6 months.

Echocardiographic Variables	Baseline	6-Months Follow-Up	*p*-Value
LVEF (%)	59.73 ± 4.55	59.24 ± 6.38	0.652
**LAVi (mL/m^2^)**	**31.54 ± 8.04**	**34.27 ± 9.99**	**0.015**
PASP (mmHg)	29.53 ± 7.54	31.64 ± 9.32	0.132
E/e’	8.14 ± 3.45	8.68 ± 3.30	0.247
GLS (%)	21.6 ± 3.10	21.52 ± 3.53	0.710
PALS 4-ch (%)	28.85 ± 9.55	27.65 ± 9.65	0.514
PALS 2-ch (%)	29.66 ± 13.74	26.50 ± 11.03	0.173
PACS 4-ch (%)	15.64 ± 6.25	14.59 ± 6.02	0.288
PACS 2-ch (%)	16.41 ± 8.16	14.64 ± 7.69	0.252

Abbreviations: LVEF, Left Ventricular Ejection Fraction; LAVi, Left Atrial Volume indexed by BSA (body surface area); GLS, Global Longitudinal Strain; E/e’, ratio of transmitral pattern; PALS, Peak Atrial Longitudinal Strain; PACS, Peak Atrial Contraction Strain; PASP, Pulmonary Artery Systolic Pressure.

**Table 3:** Comparison of baseline clinical characteristics and left atrial structural and functional echocardiographic parameters between patients who remained in sinus rhythm and those who developed atrial fibrillation during follow-up.

Clinical and Echocardiographic Variables	Sinus Rhythm Group (N = 38)	AF Group (N = 7)	*p*-Value
Current smoker	17.4%	0%	0.130
Diabetes mellitus	20.9%	28.6%	0.893
Arterial hypertension	62.5%	85.7%	0.459
Dyslipidemia	44.2%	42.9%	0.898
Peripheral vascular disease	11.6%	0%	0.357
Chronic coronary syndrome	16.3%	14.3%	0.965
LAVi (mL/mq)	31.56 ± 8.49	36.95 ± 6.98	0.116
PALS 4-ch (%)	29.68 ± 9.78	22.34 ± 14.33	0.110
PALS 2-ch (%)	29.48 ± 13.94	21.52 ± 9.05	0.183
**PACS 4-ch (%)**	**15.72 ± 6.00**	**9.75 ± 5.99**	**0.027**
PACS 2-ch (%)	16.37 ± 7.77	10.18 ± 7.37	0.072

Abbreviations: AF, Atrial fibrillation; LAVi: Left Atrial Volume index; PALS, Peak Atrial Longitudinal Strain; PACS, Peak atrial contraction strain.

## Data Availability

Data presented in this study are available on request due to privacy and ethical restrictions.
